# Supplementary material for: Delay in recovery of the Antarctic ozone hole from unexpected CFC-11 emissions
Source: Nat Commun. 2019 Dec 19;10:5781. doi: 10.1038/s41467-019-13717-x (PMC6923372; doi:10.1038/s41467-019-13717-x)
Supplement: Supplementary file 1 — Supplementary Information [file 41467_2019_13717_MOESM1_ESM.pdf]

Supplementary Information

How much longer will there be an Antarctic ozone hole? The impact of unexpected CFC-11 emissions

Dhomse *et al*.

This document provides nine additional figures which are referred to in the main text. Some further discussion is also included here.

Supplementary Results 1

A further 3-D model run is included in Supplementary Figures 1, 8 and 9 here (and Figure 6 in the main text). This run (R2000_CFC11_Ex) is similar to R2000 but uses a different future scenario of additional CFC-11 emissions related to new production. These additional emissions are similar in total to run R2000_CFC11_67 up to the year 2060 (2300 Gg, Supplementary Figure 1) but are based on large current production which is ramped down over 10 years. This production is largely accumulated in a bank which is then released at a rate 3.5%/year. Therefore, this simulation gives different time profile of emissions compared to run R2000_CFC11_67. The much larger emissions compared to run R2000_CFC11_B, even if likely unrealistic, allow us to test how the impact on ozone scales with CFC-11 emissions.

Supplementary Results 2

Supplementary Figure 1 shows accumulated emissions from different CFC-11 scenarios, with the green shading indicating the range in these emissions for the assumed uncertainties in 4 parameters (see caption). Panel (a) is annotated with the 2050 percentage recovery values and the 1980 ozone mass deficit return dates. These values can be used to estimate the impact of the assumed parameter uncertainty on these metrics. For the initial emissions, the emission ratio and the rampdown timescale the impact on the return date is around ±1 year. These contributions would combine to give a larger uncertainty, but this range will still not encompass, for example, the situation of continued non-compliance and ongoing new emissions, as implied by the WMO scenario of constant 67 Gg yr^{-1} emissions.

Supplementary Results 3

Supplementary Figure 6 further illustrates the impact of the unexpected CFC-11 emission on the Antarctic ozone hole using a 3-D model. The simulation CNTL (Supplementary Figure 6b) gives a good simulation of the observed ozone hole of 2000 (Supplementary Figure 6a). Simulation R2000 shows that by 2060 ozone recovery is expected to be well underway (Supplementary Figure 6c) with a much smaller area enclosed by the 220 DU contour. With substantial future CFC-11 emissions from renewed production (run R2000_CFC11_67) the ozone hole is still decreasing in size (Supplementary Figure 6d), but at a slower rate than R2000 (see also Figure 3). Note that run R2000_CFC11_67 assumes that production (and emission) continues at this large rate; results for simulation R2000_CFC11_B, which assumes a 10-year rampdown in production, shows much smaller differences in ozone depletion from run R2000 (Figure 4c).

Supplementary Figure 7 shows the correlation of Cly return date with October ozone return date in the Antarctic. The CTM simulations with, for example, 2000 meteorology show a compact and near-linear variation in these two quantities (e.g. panel b). Although this variation is linear it diverges from the 1:1 line as time increases. This will be due to the impact of the increasing N_2O and CH_4 scenarios in the model which cause a long-term decrease in polar ozone (see results for simulation fODS in Figure 3). For the CCMs there

is compensation from cooling of the stratosphere which increases ozone in the mid stratosphere.

Figure 6 in the main text shows the near-linear relationship of Antarctic column ozone depletion between September 21st and October 13th with accumulated CFC-11 (and CFC-12) emissions. This range of dates was chosen to match the ozone mass deficit metric used in Figure 3. Supplementary Figures 8 and 9 are similar plots to Figure 6 but means for the months of September and October, respectively. The ozone depletion for September in particular shows a strong linear correlation with the additional chlorine emission, especially over the next few decades before the influence of removal of the additional CFC-11 starts to have an impact. The variation for October, when the ozone loss rate is smaller and the vortex breaks up, is less linear.

The strong linear correlation in Figure 6, and Supplementary Figures 8 and 9 can be explained by the role of chlorine in polar ozone loss and the relative changes in the abundance of inorganic chlorine (Cly) caused by the additional CFC emissions. Polar ozone loss is dominated by catalytic cycles based on the ClO + ClO and ClO + BrO reactions. While the rate of ozone loss due to the ClO + ClO cycle will depend on $[ClO]^2$, it has been shown that the changing partitioning of ClOx (= ClO + Cl_2O_2) between ClO and Cl_2O_2 as [ClOx] changes offsets this quadratic impact. Searle et al[4] show that the effective dependence of the ozone loss due to this cycle can be $[ClOx]^{1.5}$. Furthermore, the ClO + BrO cycles, which depend linearly on [ClO], and therefore with an exponent <1 on [ClOx], make a large contribution to Antarctic ozone loss (around 30-40% over time period 2020-2040[5]). Finally, it is important to note that the Cly contributed by the additional CFC emissions is a relatively small perturbation of up to a few hundred ppt to the background Cly loading of around 3000 ppt over the next few decades. Even if a very large perturbation to Cly would cause a non-linear effect, this may not be detectable in the relatively small changes considered here.

Supplementary Figures

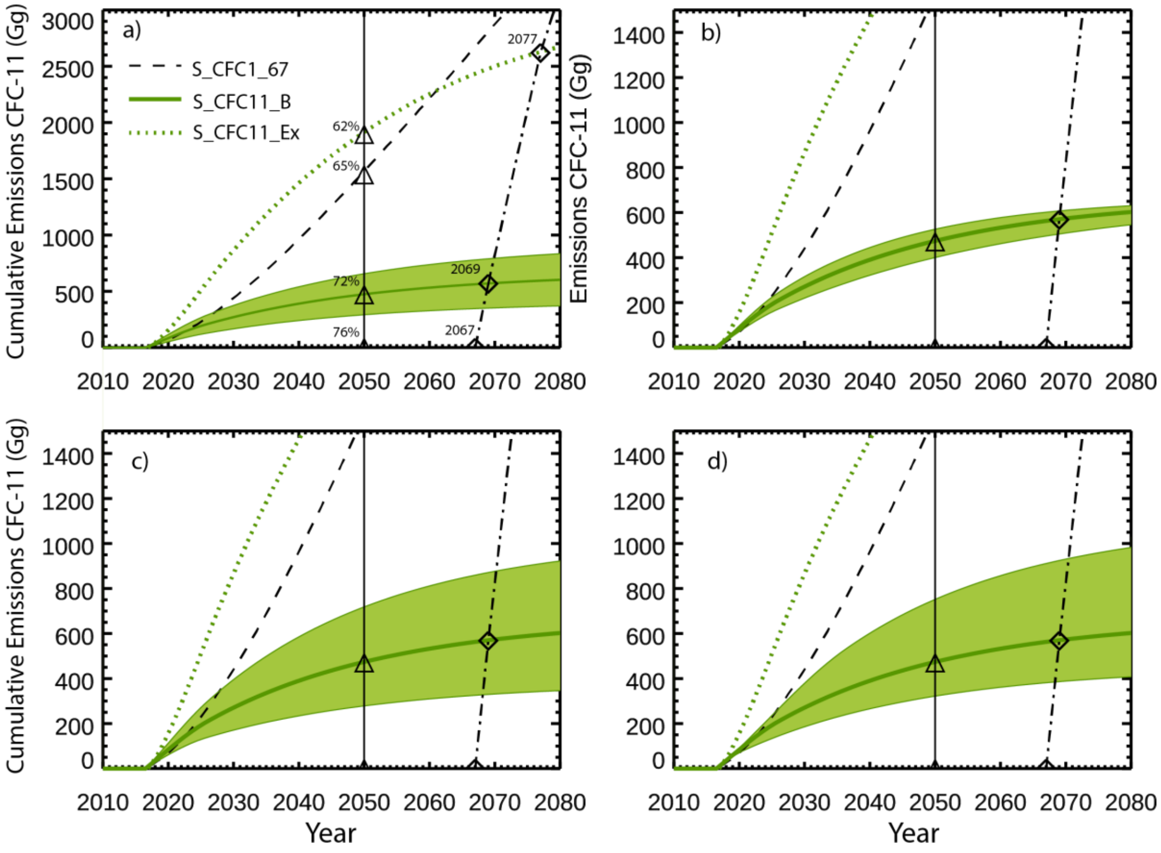

Supplementary Figure 1 | Cumulative additional CFC-11 emissions for different scenarios. The cumulative additional CFC-11 emissions (Gg) compared with WMO (2018) baseline for scenarios S_CFC11_67, S_CFC11_B and S_CFC11_Ex. The green shading shows sensitivity of the S_CFC11_B emissions to (a) initial emission rate (13±5 Gg yr^{-1}), (b) fractional release from bank (3.5±1.0 % yr^{-1}), (c) ratio of production to initial emissions (×3 - ×9, line is ×5.66) and (d) rampdown of unreported production (5-20 years, line is 10 years). The vertical line at 2050 is annotated in panel (a) with the % recovery at that date from the 3-D model runs using the given scenarios (Table 3). The dash-dot line links the 1980 return dates (annotated in panel (a)) for the 3-D model runs (Table 2). Note that the y axis range in panel (a) is double that in panels (b)-(d).

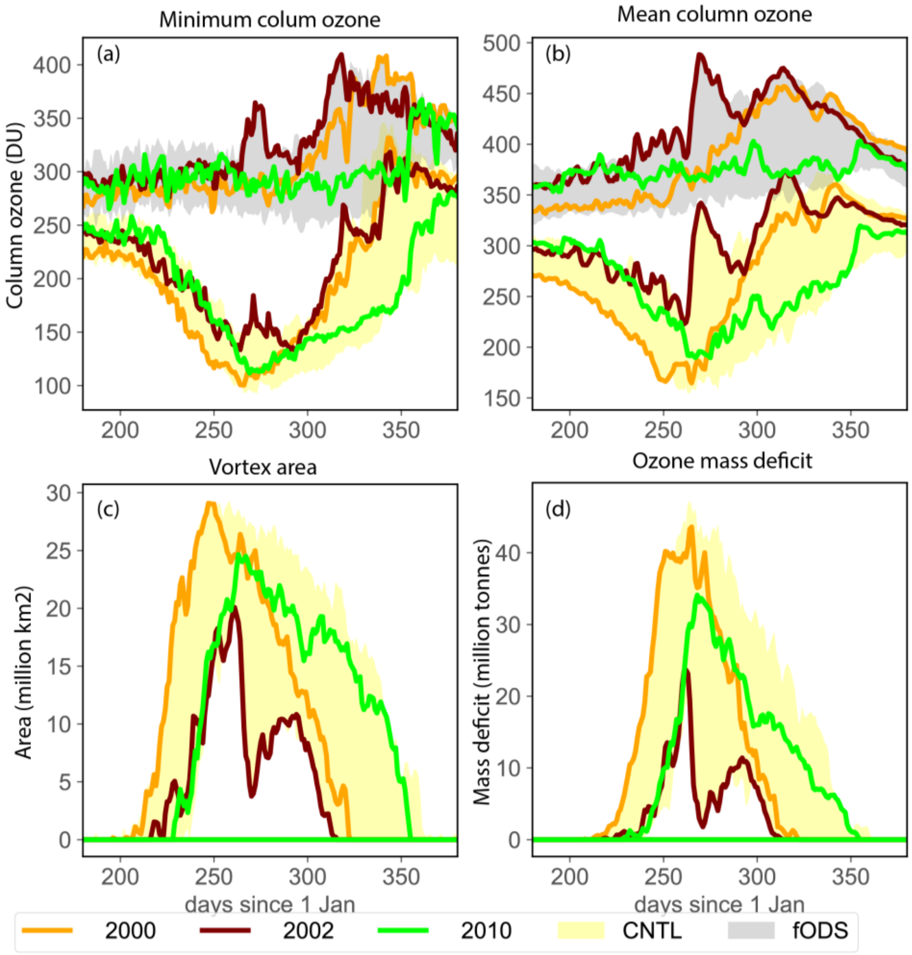

Supplementary Figure 2 | Seasonal variation of Antarctic ozone hole metrics. Seasonal variation of (a) minimum column ozone (DU), (b) mean column ozone (DU), (c) ozone hole area (×10^6 km^2) and (d) ozone mass deficit (×10^6 tonnes) for years 2000, 2002 and 2010 from model runs CNTL and fODS. The yellow and grey (panels (a) and (b) only) shading show the range of values between 1989 and 2017 from runs CNTL and fODS, respectively.

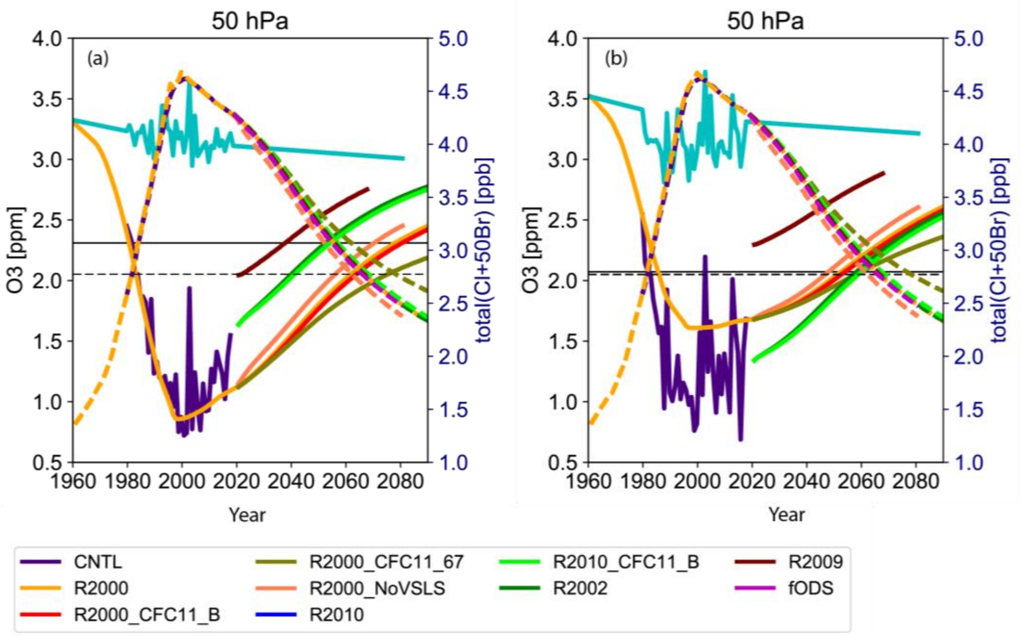

Supplementary Figure 3 | Simulated evolution of ozone and total halogens in the Antarctic lower stratosphere. Variation of ozone (solid lines, ppm) and total halogens (total chlorine + 50 × total bromine; dashed lines, ppb) from 3-D model simulations for the Antarctic (60°S – 90°S) at 50 hPa for (a) September and (b) October. The colours of the lines indicate the model experiment (see legend). Lines for the total halogen values from simulations with the same CFC-11 and VSLS scenarios overlay each other.

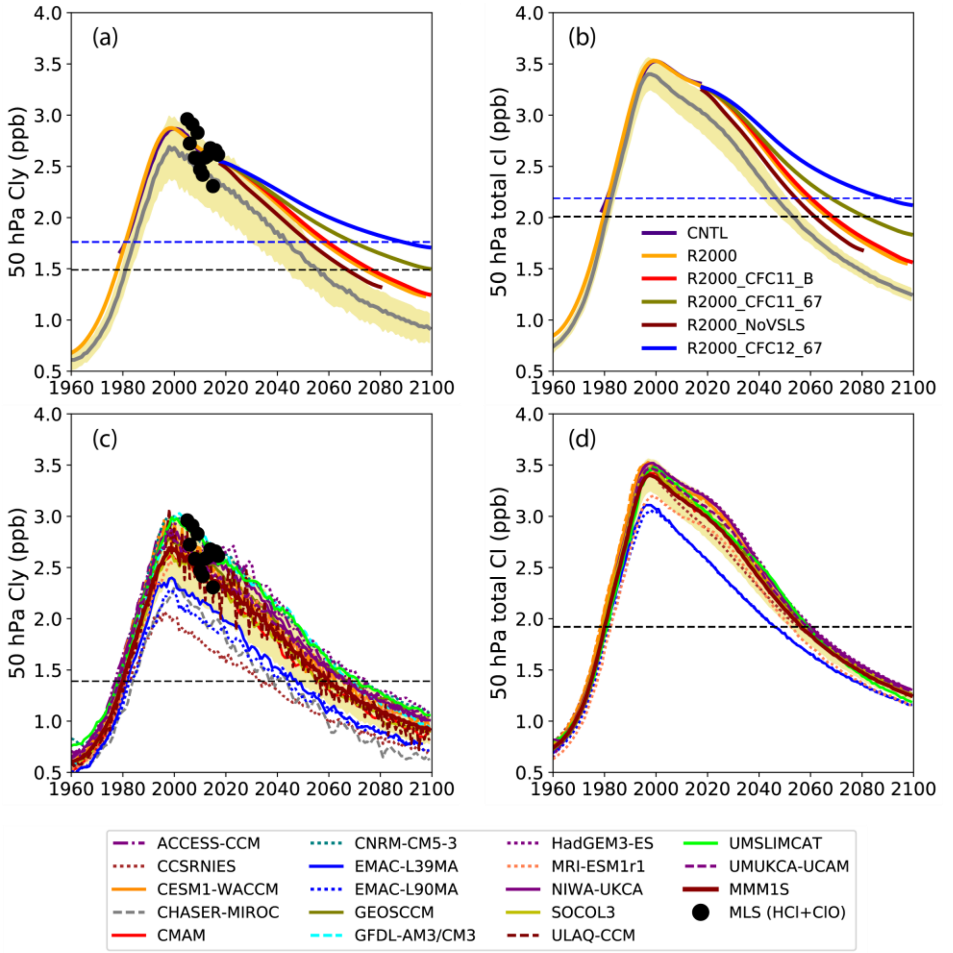

Supplementary Figure 4 | Chlorine loading of the Antarctic lower stratosphere. Evolution of (left) inorganic chlorine (Cl_y, ppb) and (right) total (organic + inorganic) chlorine from (a, b) CTM runs R2000, R2000_CFC11_B, R2000_CFC11_67 and R2000_NoVSLS, and (c, d) the Chemistry-Climate Model Initiative (CCMI) REF-C2 simulations for 15 individual CCMs and their mean (MMM1S) over the Antarctic in October at 50 hPa. The dashed black and blue (a, b only) lines show the 1980 reference values for the CCM and CTM means, respectively. The yellow shading indicates the 1σ range of the CCMs in panels (c) and (d); this range is repeated in panels (a) and (b) for comparison. Also shown in the left panels are observed October mean values of the sum of HCl and ClO from version 4 of the Microwave Limb Sounder (MLS) data[1,2] from 2005 to 2017 (black dots). The CCM panels (c, d) are taken from Dhomse et al.[3]

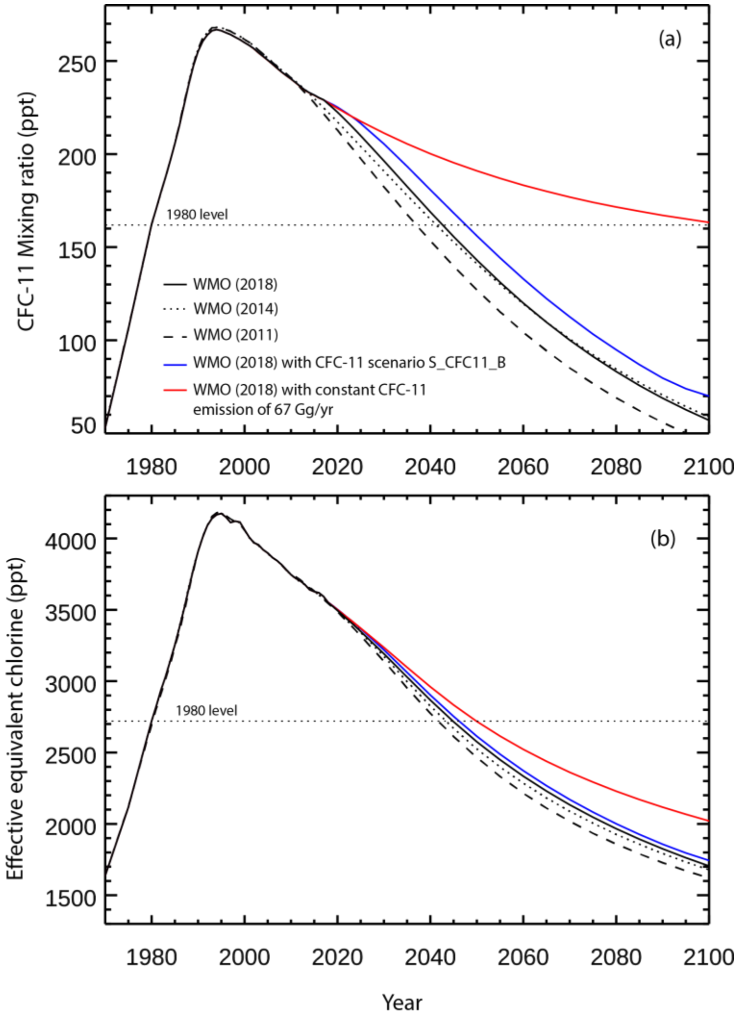

Supplementary Figure 5 | Scenarios of CFC-11 and effective equivalent chlorine. Evolution of (a) CFC-11 (ppt) and (b) equivalent chlorine (ECl, ppt) from the major ODS in the baseline scenarios in the 2011, 2014 and 2018 WMO Ozone Assessments. The horizontal dotted line indicates the 1980 CFC-11 and ECl levels. Also shown are CFC-11 scenarios S_CFC11_B and S_CFC11_67 (with constant emission of 67 Gg yr^{-1}) (see Figures 1 and 2 in main paper) and the impact of their inclusion in the WMO (2018) scenario on ECl.

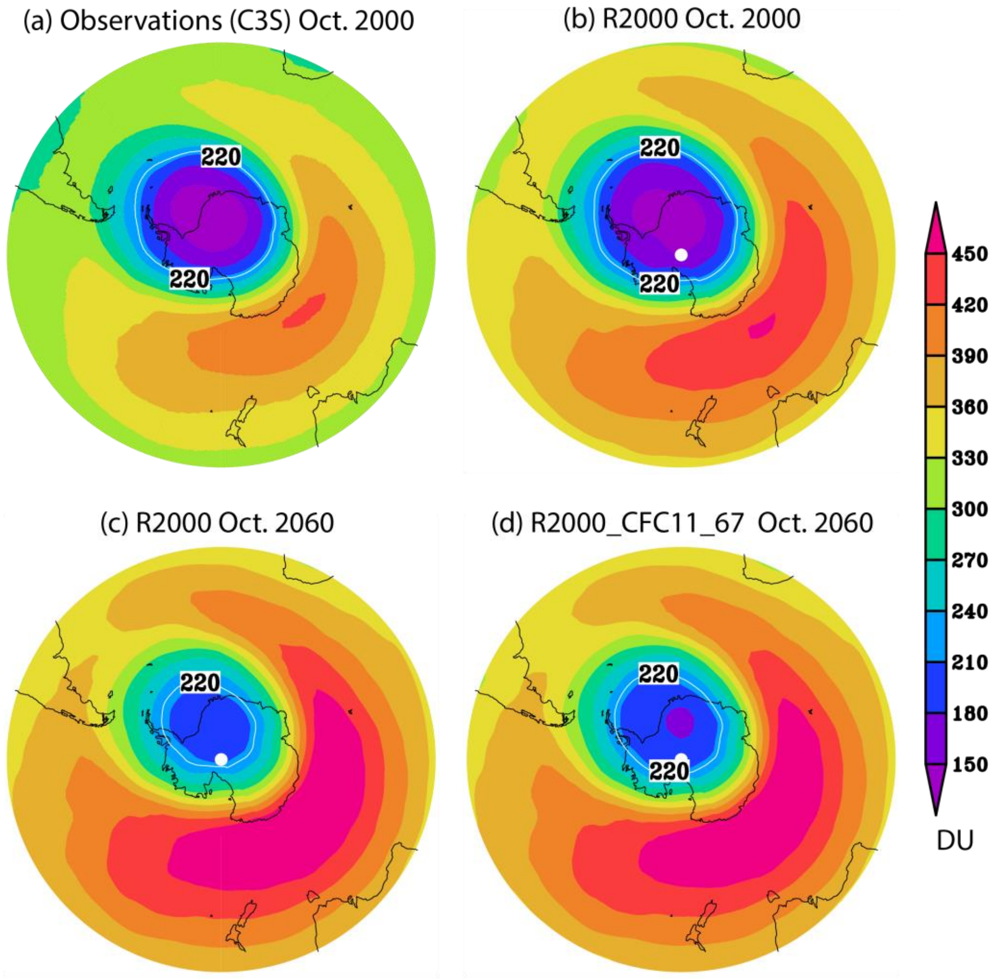

Supplementary Figure 6 | Observations and simulations of the Antarctic ozone hole showing the impact of substantial additional CFC-11 production and emission on ozone recovery. (a) Observed October mean column ozone (DU) from the C3S data product, which is based on satellite observations. Panels (b) – (d) show results from 3-D model simulations for (b) October 2000 from run CNTL, (c) October 2060 from run R2000 and (d) October 2060 from run R2000_CFC11_67. The simulations for 2060 use repeating 2000 meteorology. One measure of the size of the ozone hole is the area enclosed by the 220 DU contour, which is highlighted (Figure 3c).

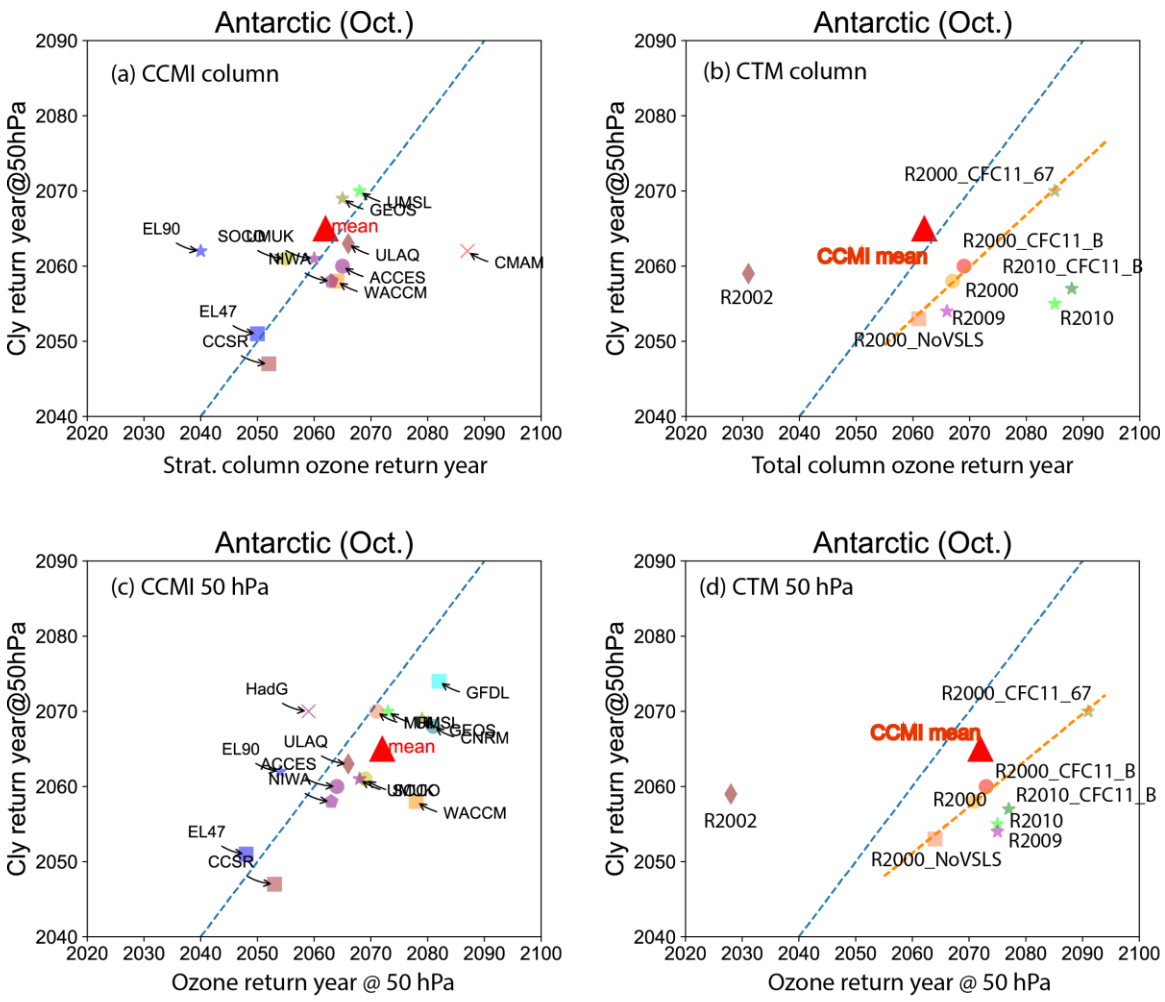

Supplementary Figure 7 | Comparison of ozone and chlorine return dates for Antarctic ozone in October. The panels show the correlation of 1980 return dates for Cly at 50 hPa with those for (a) CCMI stratospheric column ozone, (b) TOMCAT total column ozone, (c) CCMI model ozone at 50 hPa and (d) TOMCAT model ozone at 50 hPa. Panels (a) and (c) are based on Dhomse et al.[3]. Panels (b) and (d) show results from this work and the green dashed line is the fit to the simulations with the same 2000 meteorology but different chlorine loading. In all panels the 1:1 line is shown by the blue dashed line.

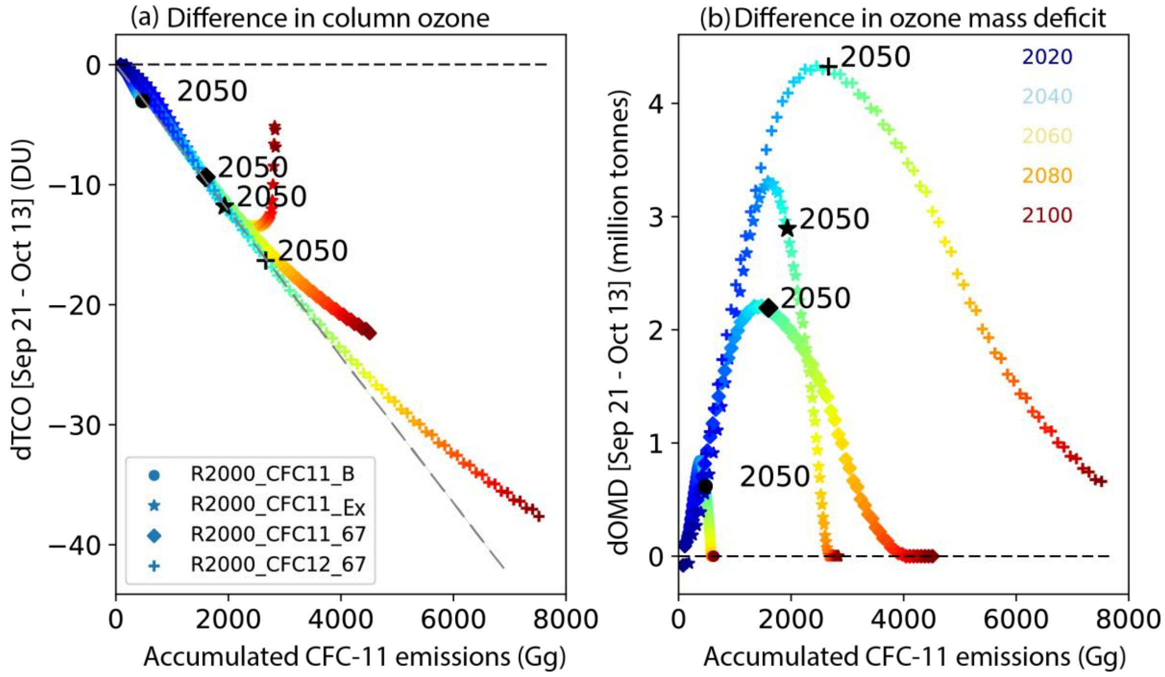

Supplementary Figure 8 | Comparison of Antarctic ozone depletion and chlorine emissions. As Figure 6 in main text but mean for September. (a) Mean column ozone difference (DU) in region 60°S-90°S for period September 1 – September 30 with respect to run R2000 for runs R2000_CFC11_B (circle), R2000_CFC11_Ex (star), R2000_CFC11_67 (diamond), R2000_CFC12_67 (+ symbol) versus accumulated additional equivalent CFC-11 emissions (Gg). The colour shading indicates the year for each data point; the points for 2050 are indicated in black. (b) Difference in estimated ozone mass deficit (million tonnes) versus accumulated equivalent CFC-11 emissions for same simulations as panel (a).

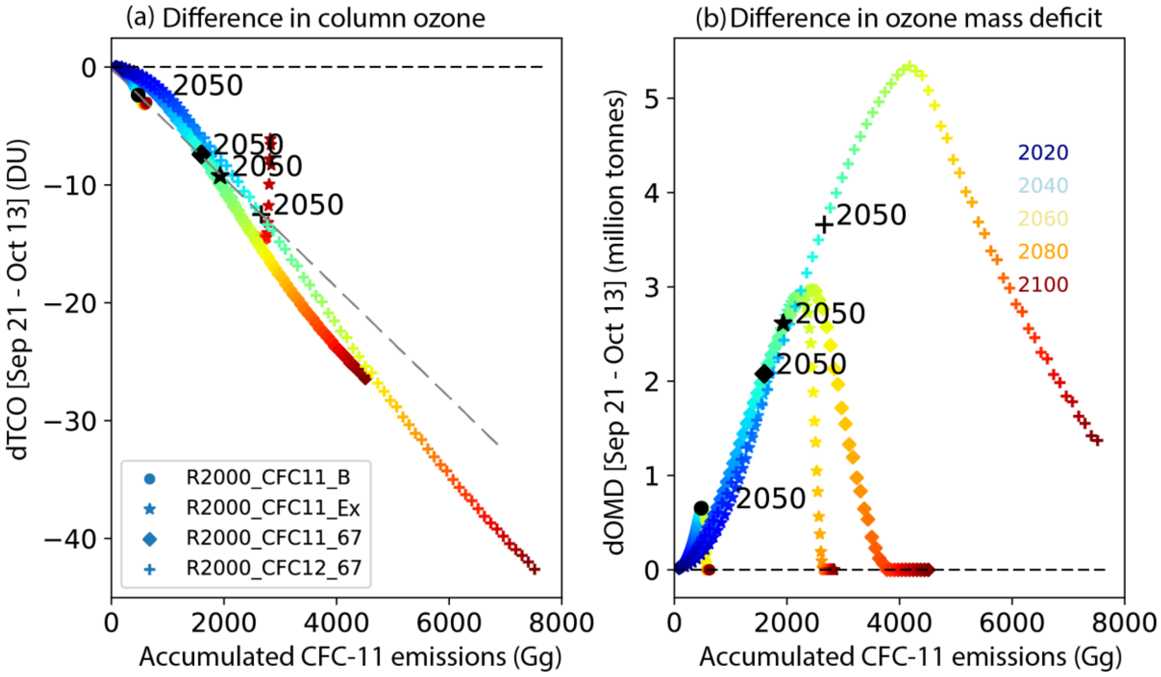

Supplementary Figure 9 | Comparison of Antarctic ozone depletion and chlorine emissions. As Supplementary Figure 8 but for October mean. (a) Mean column ozone difference (DU) in region 60°S-90°S for period October 1 – October 31 with respect to run R2000 for runs R2000_CFC11_B (circle), R2000_CFC11_Ex (star), R2000_CFC11_67 (diamond), R2000_CFC12_67 (+ symbol) versus accumulated additional equivalent CFC-11 emissions (Gg). The colour shading indicates the year for each data point; the points for 2050 are indicated in black. (b) Difference in estimated ozone mass deficit (million tonnes) versus accumulated equivalent CFC-11 emissions for same simulations as panel (a).

Supplementary References

1. Waters, J. W., Froidevaux, L., Harwood, R. S., Jarnot, R. F. & et al. The Earth Observing System Microwave Limb Sounder (EOS MLS) on the Aura satellite. *IEEE T.Geosci Remote* **44,** 1075–1092 (2006).

2. Livesey, N. J. *et al. Version 4.2x Level 2 data quality and description document*,.JPL D-33509, Jet Propulsion Laboratory, USA, https://mls.jpl.nasa.gov/data/v4-2_data_quality_document.pdf, (2018).

3. Dhomse, S. S. *et al.* Estimates of ozone return dates from Chemistry-Climate Model Initiative simulations. *Atmos. Chem. Phys* **18,** 8409–8438 (2018).

4. Searle, K. R., Chipperfield, M. P., Bekki, S. & Pyle, J. A. The impact of spatial averaging on calculated polar ozone loss: 2. Theoretical analysis. *J. Geophys. Res.* **103**, (1998).

5. Fernandez, R. P., Kinnison, D. E., Lamarque, J. F., Tilmes, S. & Saiz-Lopez, A. Impact of biogenic very short-lived bromine on the Antarctic ozone hole during the 21st century. *Atmos. Chem. Phys.* **17**, 1673–1688 (2017).
